# Interventional Treatment of Access Site Complications During Transfemoral TAVI: A Single Center Experience

**DOI:** 10.3389/fcvm.2021.725079

**Published:** 2021-11-15

**Authors:** Marcus Thieme, Sven Moebius-Winkler, Marcus Franz, Laura Baez, Christian P. Schulze, Christian Butter, Christoph Edlinger, Daniel Kretzschmar

**Affiliations:** ^1^Clinic for Internal Medicine I, University Hospital Jena, Jena, Germany; ^2^REGIOMED Vascular Center, Sonneberg, Germany; ^3^Department of Cardiology, Heart Center Brandenburg, Immanuel Klinikum Bernau, Bernau, Germany; ^4^Brandenburg Medical School Theodor Fontane, Neuruppin, Germany

**Keywords:** TAVI—transcatheter aortic valve implantation, PAD—peripheral arterial disease, interventional treatment, access site complication (ASC), pseudoaneurysms, access site bleeding, dissection, lithoplasty

## Abstract

**Introduction:** Transcatheter aortic valve implantation (TAVI) has rapidly developed over the last decade and is nowadays the treatment of choice in the elderly patients irrespective of surgical risk. The outcome of these patients is mainly determined not only by the interventional procedure itself, but also by its complications.

**Material and Methods:** We analyzed the outcome and procedural events of transfemoral TAVI procedures performed per year at our institution. The mean age of these patients is 79.2 years and 49% are female. All the patients underwent duplex ultrasonography of the iliac arteries and inguinal vessels before the procedure and CT of the aorta and iliac arteries.

**Results:** Transfemoral access route is associated with a number of challenges and complications, especially in the patients suffering from peripheral artery disease (PAD). The rate of vascular complications at our center was 2.76% (19/689). Typical vascular complications (VC) include bleeding and pseudoaneurysms at the puncture site, acute or subacute occlusion of the access vessel, and dissection or perforation of the iliac vessels. In addition, there is the need for primary PTA of the access pathway in the presence of additional PAD of the common femoral artery (CFA) and iliac vessels. Balloon angioplasty, implantation of covered and uncovered stents, lithoplasty, and ultrasound-guided thrombin injection are available to treat the described issues.

**Conclusion:** Interventional therapy of access vessels can preoperatively enable the transfemoral approach and successfully treat post-operative VC in most of the cases. Training the heart team to address these issues is a key focus, and an interventional vascular specialist should be part of this team.

## Introduction

Transcatheter aortic valve implantation (TAVI) has evolved rapidly over the past decade and is now the treatment of choice in the patients over 70 years of age, regardless of surgical risk (1). The outcome of these patients, especially at the low surgical risk, is essentially determined not only by the interventional procedure itself, but also by its complications (2). In that context, the specific access site and the associated risk of complications are still a matter of debate (3).

The transfemoral approach is associated with a number of potential challenges and complications. The rate of major vascular complications, such as relevan bleeding events range from 3.5 to 9.3%. Thus, the probability of such complications is significantly higher than the rate of access site complications requiring treatment after the peripheral interventions, which is 3–4% (4, 5). When considering only life-threatening bleedings, there is a frequency of 2–2.6% during or directly after the TAVI procedures. The occurrence of major VCs is associated with a significantly increased 30-day mortality (6–8). An ultrasound-guided puncture has the potential to significantly reduce the rate of access site complications (9, 10).

Typical complications of the transfemoral vascular access route are hemorrhage and pseudoaneurysms at the puncture site, acute or subacute occlusions of the access vessel, usually the common femoral artery (CFA), and dissections or perforations of the iliac vessels (6, 7).

Since etiological risk factors of aortic stenosis overlap with those of classic atherosclerosis, such as PAD, the coincidence of these diseases is common. In large Medicare databases, this coincidence ranges between 24.5 and 49.7% (11). Although TAVI abort rates have steadily decreased from 4 to 1% in recent years, mainly due to technological improvement of the devices and delivery systems, PAD is still the most important risk factor for failure (odds ratio [*OR*] 1.88, *p* < 0.01) (12) and an independent predictor of mortality (13). Not only against the background of multimorbidity and/or the advanced age of the patients with TAVI, an interventional therapy of the above-mentioned complications is preferable compared with the classical surgery (14, 15).

There is still a relevant number of cases, in which the transfemoral access route need to be considered unsuitable for primary TAVI access because of heavy calcifications, high-grade-stenoses, and occlusions of the CFA or iliac vessels (12). For these situations, the central approaches (transapical or transaortic) as a surgical alternative and the non-femoral approaches (trans-carotid, trans-axillar, and trans-subclavian) are an option. Since the rate of complication when using the non-femoral vascular approaches is not inconsiderable and their occurrence is associated with an increased mortality (16), preoperative conditioning of the transfemoral approach by the vascular interventions is definitely an attractive option that is favored not only by our center (17).

We aimed to characterize the specified therapeutical strategies preferred in our department for the typical issues and complications.

## Materials and Methods

Between 2017 and 2020, 689 transfemoral TAVI procedures were performed at our institution. The mean age of these patients was 79.2 years and 49% were female. The mean Society of Thoracic Surgeons (STS) risk score was 4.5%. Short-term (30 day) mortality ranged between 2.0 and 2.5% per year in this period of time. All the patients underwent duplex ultrasonography of the iliac arteries and inguinal vessels before the procedure and CT of the aorta and iliac arteries.

Usually, one femoral access was used for the necessary large-lumen sheath, and an additional femoral or radial access was created. After sheath insertion, the correct positioning was documented angiographically. In the case of preoperative findings suggesting difficult puncture or in known CFA stenoses, an ultrasound-guided puncture was selected.

No routine angiography of the puncture site was performed; only in the cases of difficulties before or after removal of the large-lumen access, the pelvic axis and/or the puncture site were visualized by angiography in a cross-over or transbrachial approach *via* the already existing second access. The vascular complications were immediately treated interventionally to avoid the open vascular surgery in this patient population.

## Results

The rate of vascular complications was 2.76% (19/689), such as common femoral artery aneurysms (*n* = 17), dissections (*n* = 2), and severe bleedings (*n* = 7); one patient showed aneurysm and dissection and six patients showed aneurysm and severe bleeding. All the patients underwent interventional attempt to resolve the complications related to the access vessels, which occurred during the TAVI procedure, or as barriers to a planned TAVI as well. In the following, the typical complications and obstacles encountered during or before the TAVI procedures are mentioned, and interventional strategies that have proven helpful in the named complications are presented.

### Bleeding Complications

#### Postoperative Bleeding at the Puncture Site

Bleeding at the puncture site may occur as early after puncture or sheath insertion due to plaque-rupture but becomes relevant most commonly at the end of the TAVI procedure due to failure of the vascular closure system. This is to be expected especially in the heavily calcified vessels. In some cases, the bleeding occurs for some time after TAVI, for example, with the appearance of a retroperitoneal hematoma or malfunction of the vascular closure device.

**Figure 1:**
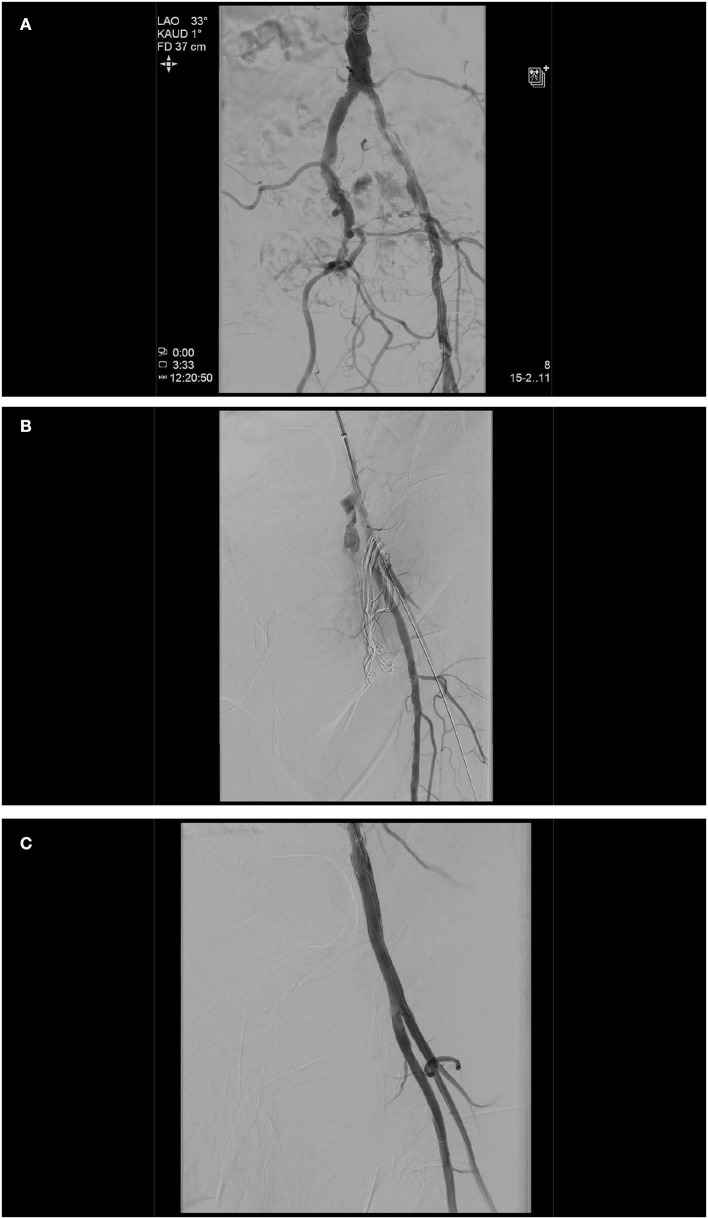
Initial angiogram demonstrating severely calcified left iliac arteries and a chronic occlusion of the right external iliac artery **(A)**. As a second arterial access, the left brachial artery was chosen, based on the fact that only one brain-supplying artery (left vertebral artery) would be crossed in the process, the shorter and more direct route to the peripheral arteries in the event of an access site or a problem even further downstream. A transcatheter aortic valve implantation (TAVI) procedure itself was done without any complications (26 mm Edwards Sapien 3, Edwards, CA, USA). At the end of the TAVI-procedure, as expected, it was not possible to close the left femoral access by Perclose ProGlide™ (Abbott, IL, USA) knot systems. Angiography displayed access site bleeding **(B)**. We changed the left brachial artery sheath to a 6 F 100 cm sheath (Fortress®, Biotronik, Germany), blocked common femoral artery with a 6.0 mm × 60 mm balloon (Mustang™, Boston Scientific, MA, USA), and sealed bleeding with a 6.0 mm × 50 mm covered stent prothesis (Gore® Viabahn® Endoprosthesis, Gore, DE, USA) **(C)**.

A cross-over approach or a transbrachial approach has proven to be effective in this case, allowing angiographic control of the success of the vessel closure system and immediate treatment of any failure. In the cases, where we anticipate problems with the vascular closure device, we primarily prepare this additional access. As first therapy, the bleeding must be stopped by inflation of a 6–8/40 mm PTA balloon, should this not be sufficient, followed by the implantation of a covered stent (Figure 1).

#### Pseudoaneurysms at the Puncture Site

**Figure 2:**
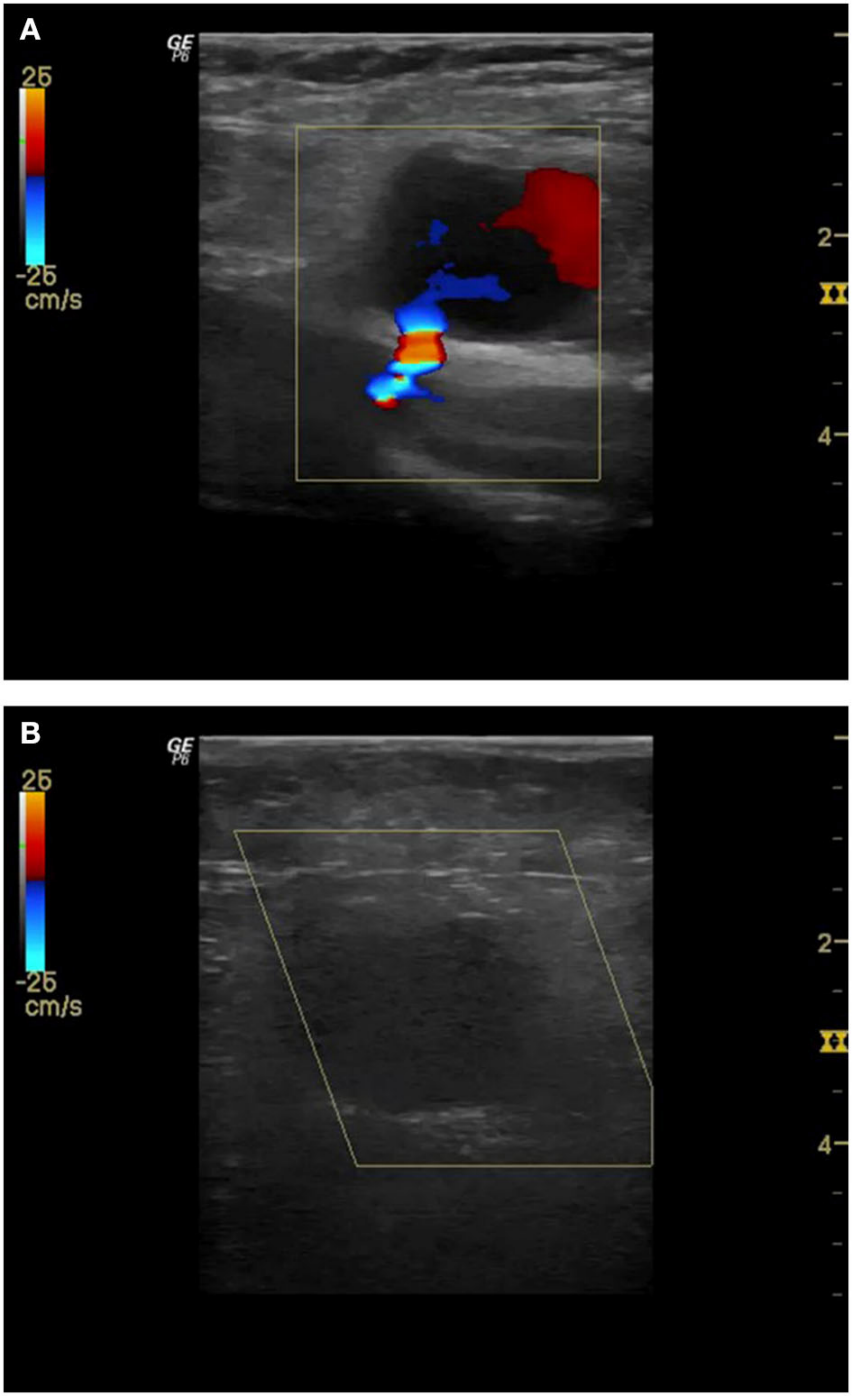
Pseudoaneurysm with clearly visible bleeding from common femoral artery (CFA) after TAVI **(A)**. Since compression with the ultrasound-probe shows no success even after 30 min and an ultrasound-guided injection of 500 IU thrombin is performed. Subsequently, complete closure of the pseudoaneurysm **(B)**.

Pseudoaneurysms usually present after removal of the compression bandage. They may manifest as pain, a swelling in the groin, or as hematoma, but may also remain clinically invisible. Therefore, all the patients at our clinic receive a duplex sonographic check of the groin on the following day. Therapy of choice is the first attempt of ultrasound-guided compression. If this does not lead to success, which is common with large lumen accesses, the local thrombin injection under duplex sonographic control is our next step. Only in the cases of very pronounced hematoma and skin lesions, surgical therapy is necessary (Figure 2).

#### Perforation of the Iliac Vessels

Perforations of the iliac vessels may occur at any time during passage with the device, especially in the presence of severely calcified vessels or stenoses. Repeated angiographic control is recommended in case of strong resistance of the pelvic passage, circulatory sensations, and after removal of the device.

**Figure 3:**
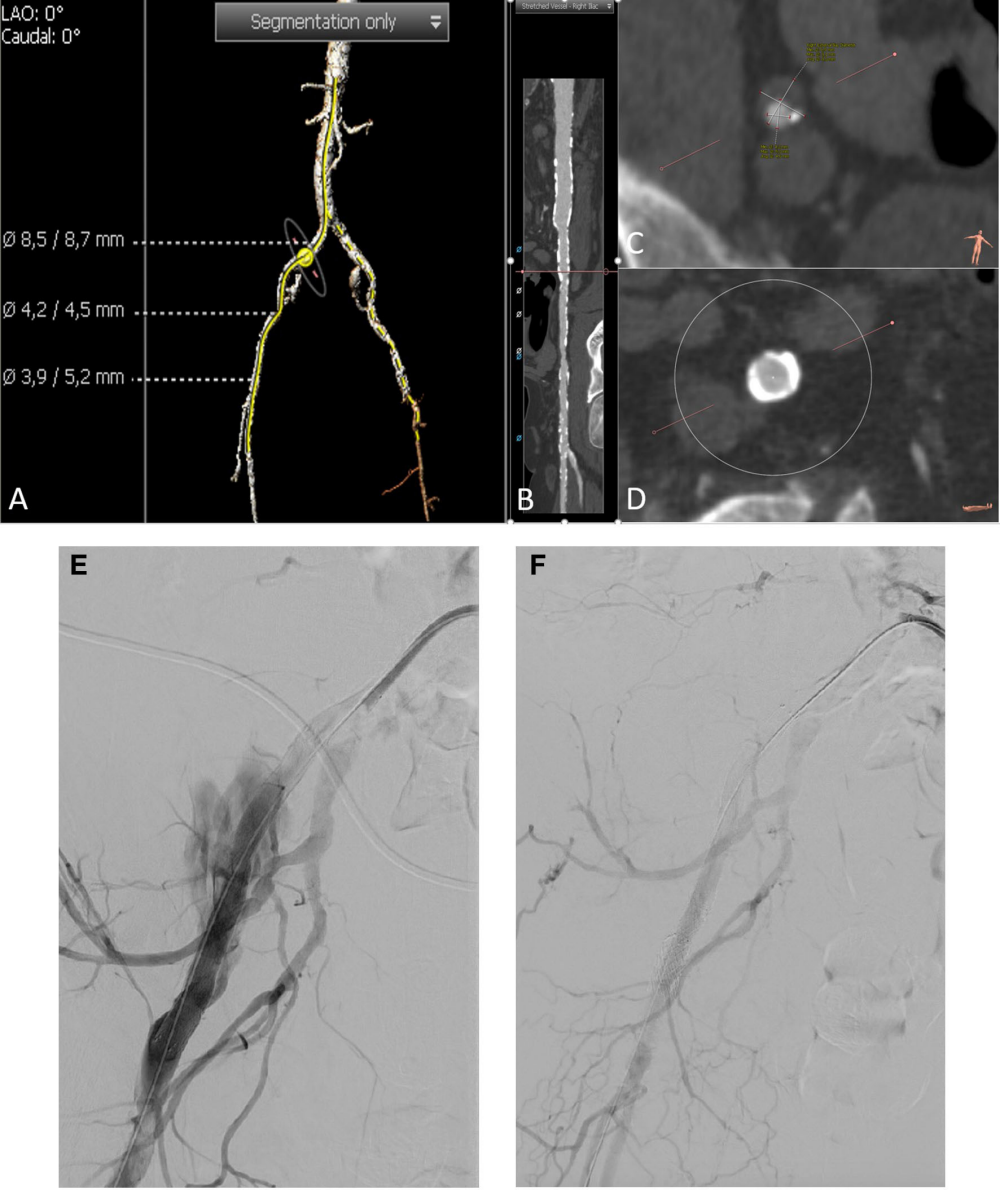
A CT angiography unraveled severely calcified iliofemoral arteries **(A–D)**. Three-dimensional reconstruction of pelvic arteries with the measurement of common femoral artery diameter, minimal external iliac diameter, and common femoral arterial diameter (from up to down) **(A)**. A stretched vessel analysis of the right pelvic axis shows severe calcification of the vessel **(B)**. Minimal diameter of the right external iliac artery is 4.2 mm **(C)**. Right common iliac artery with circular calcification **(D)**. After initial discussion of a pre-TAVI vessel preparation with by lithoplasty according to the CT angiography, we decided to go on without any peripheral intervention before TAVI. The balloon valvuloplasty of the aortic valve was performed without any complications. Afterward, we were not able to pass the new valve (CoreValve™, Medtronic, Ireland) through the iliac artery on the right side. Thus, we decided to upsize the sheath to 18 F (Sentrant™, Medtronic), again we were not able to pass the iliac arteries. Another angiography showed a perforation of the right external iliac artery **(E)**. We immediately blocked the bleeding site with an 8.0 mm × 80 mm balloon (Charger, Boston Scientific, MS, USA). We now implanted an 8.0 mm × 100 mm as well as a 8 F compatible 11 mm × 39 mm balloon expandable covered stent prothesis (Gore® Viabahn® VBX, Gore) which finally fixed the perforation **(F)**.

In the case of perforations of the pelvic arteries, an immediate balloon obstruction of the feeding vessel or in case of very proximal problems also of the aorta must be performed, the required material must be available in the catheterization laboratory. For definitive treatment, a covered stent or, at the level of the aorta, an aortic prosthesis will be implanted (Figure 3).

### Occlusions and Dissections

#### Occlusion of the CFA After the Procedure

Occlusions of the CFA usually occur after placement of the vascular closure devices (VCD), especially if needle- or needle-based systems [Perclose ProGlide™ (Abbott)] are combined with collagen-based systems [e.g., AngioSeal (Terumo, Japan)]. In addition, the advancement of large-lumen devices can set up plaques which then occlude the vessel lumen. The occlusion manifests as acute or subacute ischemia, which should be noticed before the patient is removed from the catheterization laboratory. A clinical check of the foot of the punctured leg and a doppler-ultrasound on the following day are essential.

**Figure 4:**
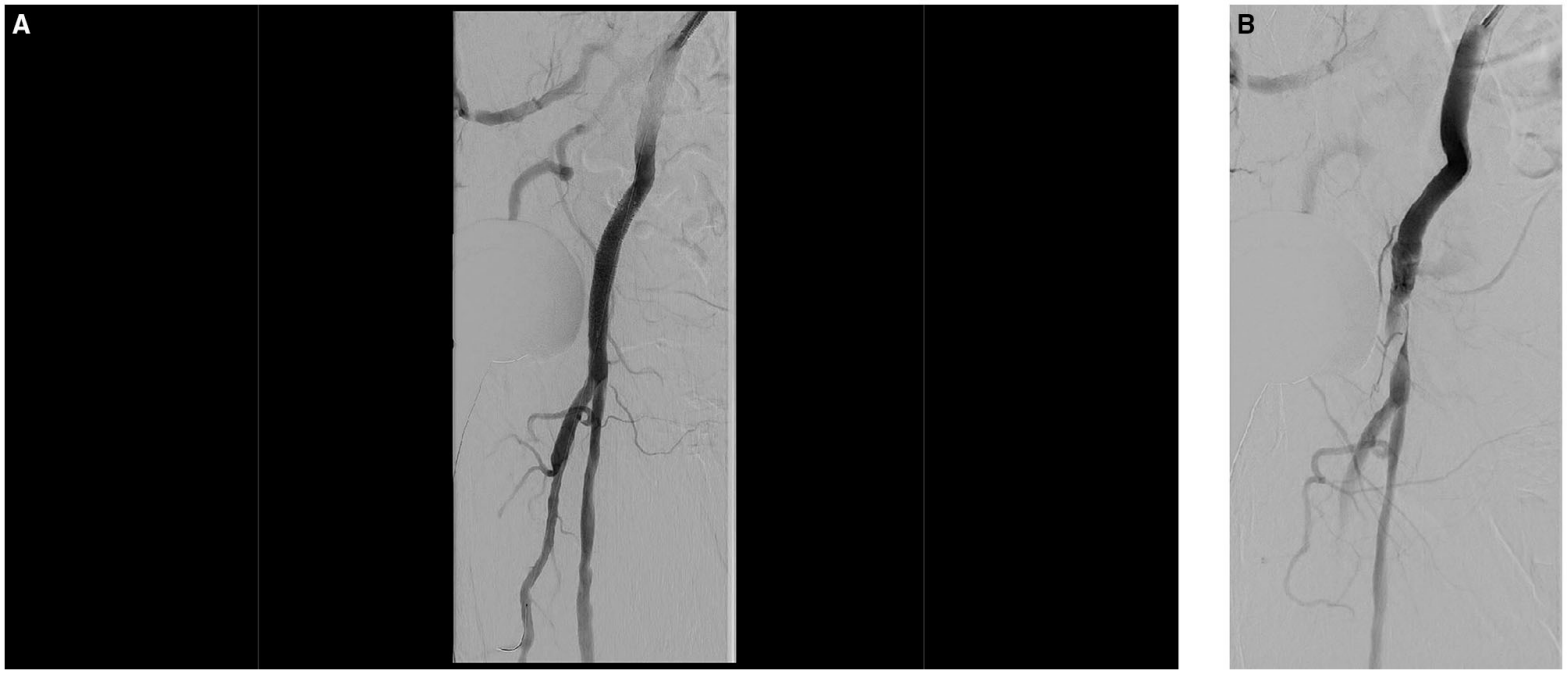
The valve was implanted without any complications (26 mm Evolut R, Medtronic), using a 14 F sheath on the right and a 6 F sheath on the left side. Access to the right femoral artery was closed using two previously administered Perclose ProGlide™ (Abbott) systems. Control angiography showed delayed contrast flow in the right ileofemoral axis and was therefore suggestive for an acute occlusion of the right femoral artery. The crossover angiogram confirmed a femoral artery occlusion and, in addition, a small bleeding **(A)**. Left femoral sheath was changed to 6 F 45 cm (Fortress®, Biotronik) sheath. Occlusion was passed with a 0.0018-inch wire (Glidewire Advantage®, Terumo) and dilated using a 4.0 × 40 mm balloon (Passeo-18, Biotronik). Subsequently, we implanted an 8.0 × 50 mm covered stent prothesis (Gore® Viabahn® Endoprosthesis, Gore). Due to the necessity of implanting a stent in the highly flexible femoral artery we decided us to stabilize this by implantation of an interwoven 7.5 × 40 mm stent (Supera™ Stent, Abbott). Final angiography displayed a very good result **(B)**.

If the thrombotic occlusions are caused by the VCDs, suction catheters usually do not help. If necessary, a rotational thrombectomy can be considered, but in this case the distal dislocations of the anchor of the VCD are common. In our opinion, the safest and simplest method is the implantation of a covered stent. To avoid later problems due to kinking in the CFA, we like to combine with an interwoven Supera™ stent (Abbott) (Figure 4).

#### Dissection of the Iliac Arteries

**Figure 5:**
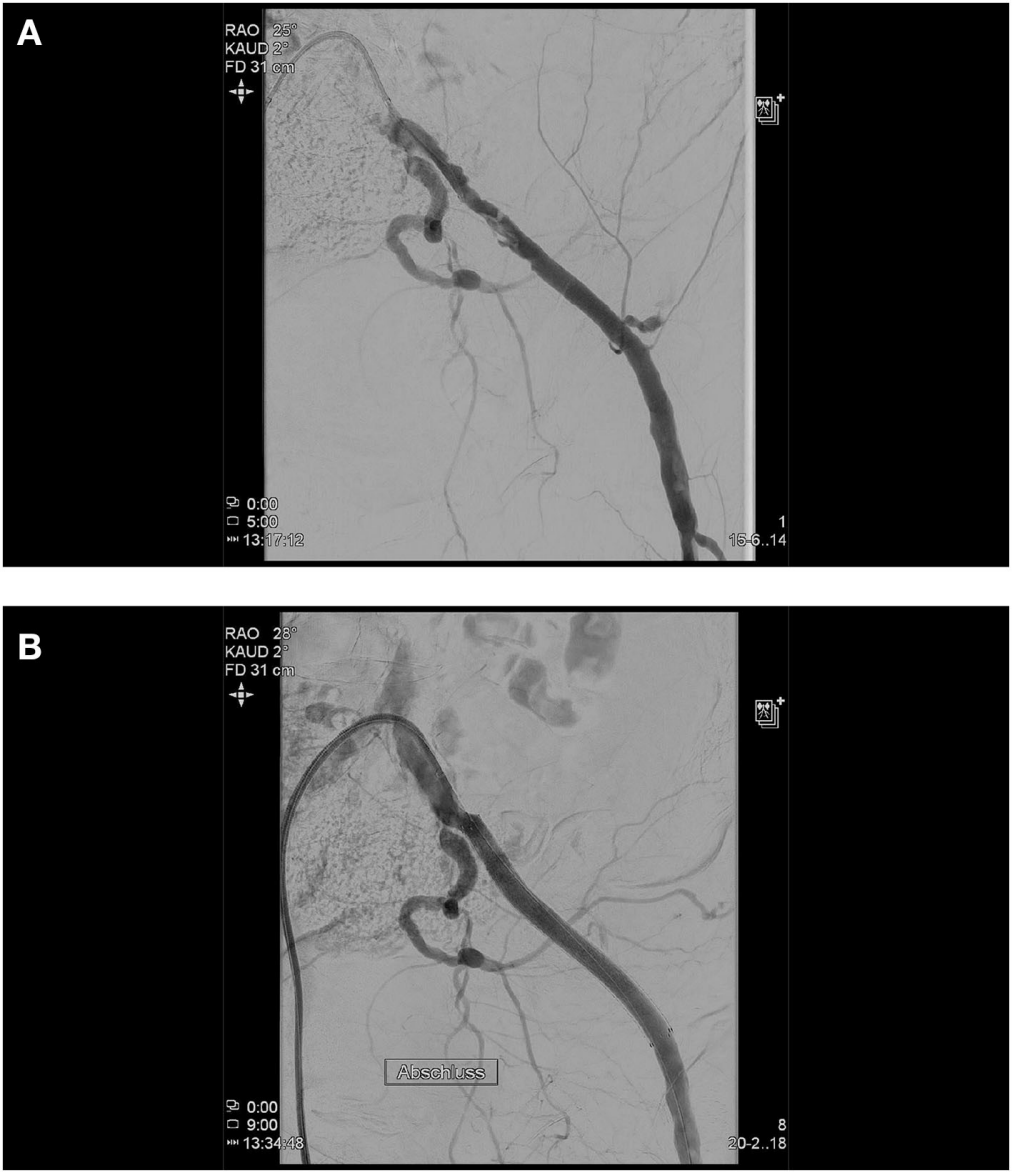
During the TAVI procedure, both the femoral arteries were punctured and cannulated with a 5 F sheath left and a 14 F sheath on the right side. After successful implantation of the TAVI device, right femoral puncture site was successfully closed using two previously administered Perclose ProGlide™ (Abbott) systems. The 5 F sheath on the left side was removed and after manual compression for approximately 10 min with no overt bleeding, the lady got a compression bandage and was transferred to our immediate care unit. After 30 min, she developed an acute ischemia of the left leg, which improved after relaxation of the pressure dressing. Then, the lady developed a hemorrhagic shock and was set on volume expansion and inotropes. The immediate right femoral crossover angiography shows, in addition to a dissection of the left external iliac artery, an active bleeding from the left hypogastric artery caused by the initial puncture and the sheath **(A)**. Using a 45 cm 7 F sheath (Destination Guiding Sheath, Terumo), the bleeding site was blocked utilizing an 8.0 mm × 40 mm balloon (Mustang™, Boston Scientific) and an 8.0 mm × 100 mm covered stent prothesis (Gore® Viabahn® Endoprosthesis, Gore) was implanted which sealed both, the hypogastric bleeding and external iliac dissection **(B)**.

The iliac artery dissections may occur during wire passage or device passage. It is important to recognize these and treat them appropriately, even though they need not result in a clinically relevant problem during the procedure. A final angiography of the iliac arteries is therefore necessary from our point of view. The standard therapy is implantation of uncovered nitinol stents or, if thrombus formation is suspected, covered stents (Figure 5).

### Preexisting Stenoses and Occlusions of the CFA and Iliac Vessels

**Figure 6:**
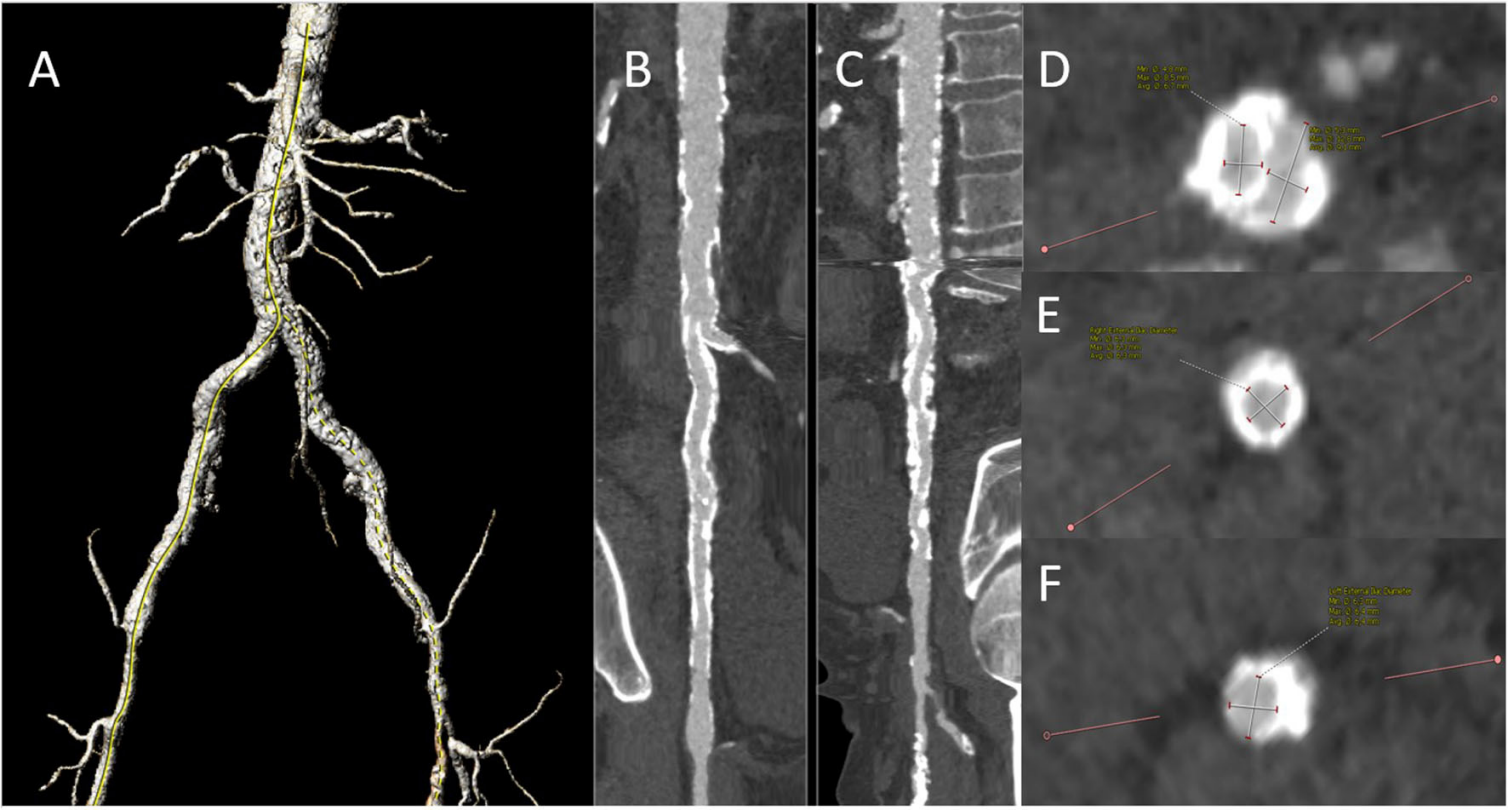
Preinterventional CT angiography demonstrating severe aorto-iliac PAD: 3-D reconstruction of pelvic vessels **(A)**. A stretched vessel analysis of the right **(B)** and left **(C)** axis and infrarenal aorta demonstrating severe calcification. Slice of the iliac bifurcation **(D)**, the right external iliac artery **(E)**, and the left external iliac artery **(F)** Both the iliacs were already provided with the self-expandable stents in the past. Duplex ultrasound displayed hemodynamically severe iliac stenoses with a peak systolic velocity of 2.6 m/s in the right common iliac and 3.0 m/s in the left external iliac artery. We decided to perform a staged procedure with the preparation of the iliac vessels first.

**Figure 7:**
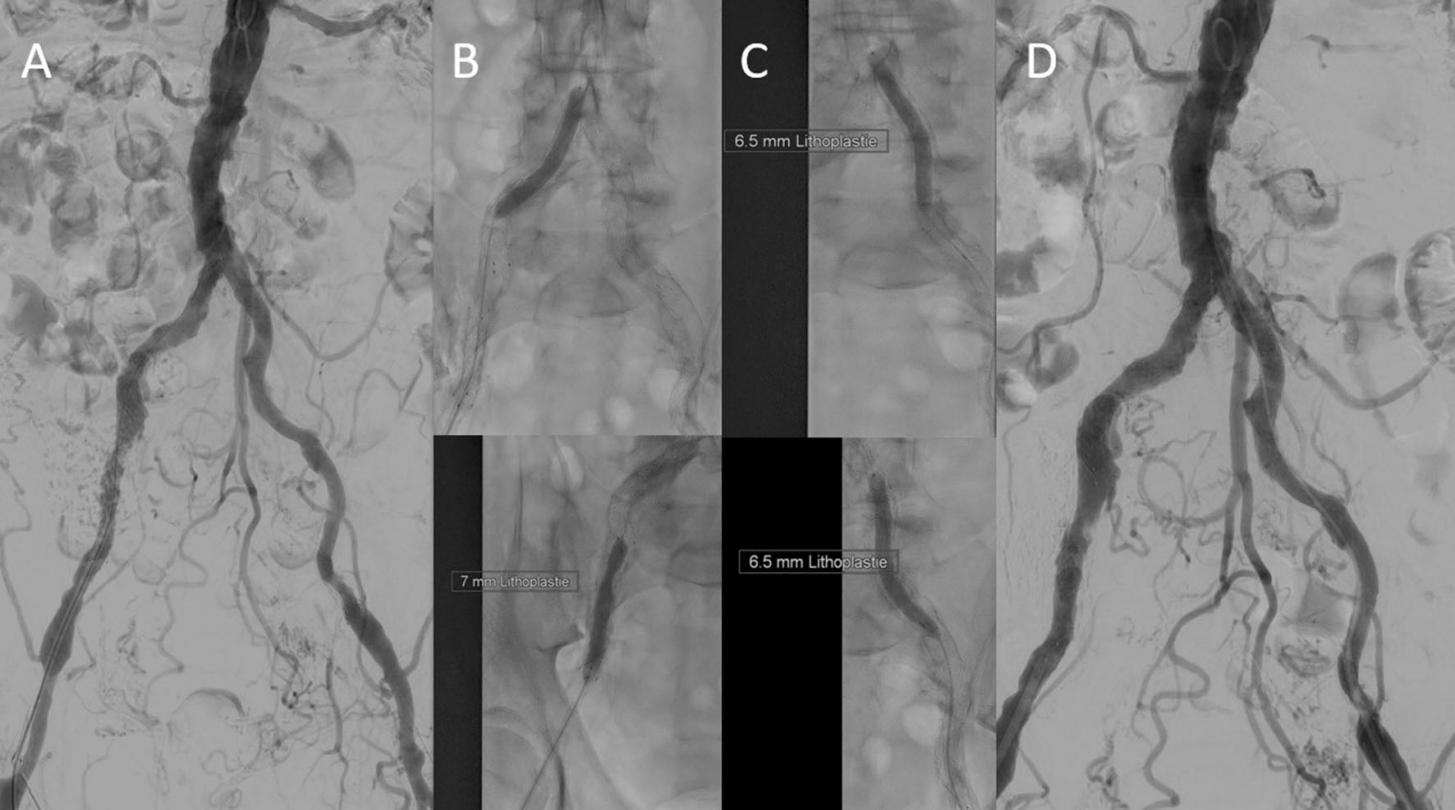
Initial digital subtraction angiography confirmed severe calcification. **(A)** The right iliac arteries were dilated using a 7.0 mm × 60 mm intravascular lithoplasty (IVL) device (Shockwave Medical, Inc., CA, USA) **(B)**, after three cycles (90 pulses), the device ruptured. This was due to a malapposition of a distal stent strut in the external iliac artery. The left iliac axis was treated with five cycles (150 pulses) using a 7.0 mm × 60 mm IVL balloon (Shockwave Medial, Inc.) **(C)**. The result was satisfying **(D)**. Transfemoral TAVI (26 mm Edwards Sapien 3, Edwards) was done 6 days later by using a 14 F sheath *via* left femoral access. Nevertheless, after successful TAVI, the need for covered stent prosthesis implantation to fix external iliac artery perforation was necessary due to the heavy calcification.

Due to the frequent prevalence of stenosis of the access vessels in the patients with indicated TAVI, a duplex scan must regularly be performed before the intervention, and often an additional CT angiography of the iliac arteries (Figure 6). This allows us to decide whether a puncture, for example, proximal to a detected stenosis, is possible or whether a pretreatment of the vessel is necessary. We have made very good experiences with lithoplasty in highly calcified vessels both in the CFA and in the iliac arteries, where a complete expansion of the implanted stents is necessary to allow the passage of the device (Figure 7). In addition, the large-lumen stents must be chosen, which may still require post-dilatation. The covered stents offer a valid alternative. Whether the intervention is performed a few weeks before the TAVI procedure or at the same time depends on the priority of the TAVI.

## Discussion

The evolution of TAVI represents one of the greatest innovations in the clinical cardiology in recent decades (18, 19). Since the indications for TAVI have steadily been expanded in recent years and that the method is mainly applied in the high-risk patients, knowledge about complication management is of certain interest (20). In that context, vascular access has always played a central role. Increasing clinical expertise with the TAVI technique and the development of novel devices and delivery systems has already led to a decrease in the complications. Nevertheless, vascular access still represents an “Achilles' heel” of treatment, especially in the patients with incidental PAD. The outcome of these patients is essentially determined not only by the interventional procedure itself, but also by its complications (6–8).

In the current literature, the rate of major vascular complications or bleeding is reported to be as high as 3.5–9.3% for transfemoral TAVI access patients. A clear association of their occurrence with an increased 30 day mortality could be demonstrated in recent years not only in the high-risk patients (6–8). Furthermore, it can usually be assumed, that the patients with a serious vascular complication will have to endure a longer hospital stay and postoperative mobilization time. As many of the cases are the geriatric patients with numerous comorbidities, the prolonged hospital stay carries the risk of, among others, nosocomial pneumonia. In addition, It is known, that delayed mobilization has a negative impact on the quality of life and might have an impact on the geriatric assessment scores of mobility and autonomy of the patients (21–23). Large hematomas are the potential sources of infection also, which is very unpleasant during a recently performed valve intervention and may have to be treated with the continued administration of perioperative antibiotics.

An emergency vascular surgery required after the TAVI procedures significantly worsen the patient outcome (6, 7). Although the choice of a non-femoral approach, such as trans-carotid, trans-axillar, or trans-subclavian, reduced the number of emergency vascular surgeries, it did not improve the overall complication rate (16). Therefore, the focus must be on the rapid and safe treatment of the VCs, preferably with less invasive methods.

Using the methods presented above, we are able to demonstrate, that in the typical cases of VCs, such as dissections and perforations of the iliac vessels and postoperative bleeding or occlusion of the puncture site, an interventional treatment can be performed successfully, safely, and with minimum discomfort for the patient. In all the reported cases, we would therefore recommend an interventional procedure. Nevertheless, the involvement of surgical expertise is essential in all these cases, because there will be patients, in whom the surgical therapy, such as hematoma evacuation, suturing, endarterectomy with patch of the common or superficial femoral artery, bypass surgery or hybrid approach will be required (24, 25).

In the case of VCs forecasting problems becoming obvious during the TAVI procedure, a 0.018″ wire is inserted to the access side from the contralateral groin as a safety wire into the distal SFA. This allows visualization of the iliac vessels from contralateral before removal of the large lumen sheath and the necessity of interventional complication management can be determined. In all the cases, angiographic imaging of the puncture site should be performed after the insertion of a first small caliber sheath (ipsilateral 30° angulation).

However, general prophylactic endovascular protected sealing of the access vessel does not provide any benefits, as shown in a large collective very recently (8). Since it may even increase the rate of minor VCs and acute renal failure, we do not generally recommend this approach and suggest its use only in the cases of proven need. In addition, the new vascular closure devices, such as MANTA® or PerQSeal®, specifically designed to close the large bores at the femoral arterial access sites, may further reduce the complication rates (26). In a small group of 311 patients, a significantly reduction of all-cause mortality (0% vs. 4%, *p* = 0.02), vascular (14% vs. 21%, *p* = 0.21), and bleeding complications (18% vs. 33%, *p* = 0.01) could be observed in MANTA® Vascular Closure Device- vs. ProGlide-treated patients (27).

In addition, it is important to keep in mind the specific problems of the patients with preexisting concomitant PAD. This issue is of special concern since the number of elderly aortic stenosis patients with diabetes mellitus, renal failure, and PAD eligible for TAVI treatment is expected to continuously increase in the near future. Especially in these cases, we were able to demonstrate that a vascular intervention, ideally scheduled prior to TAVI, is usually capable to facilitate the femoral access for the TAVI procedure. The patients in whom an iliac stent is necessary, we recommend, whenever possible, postponing the TAVR procedure until the stent has healed to avoid the stent dislocation through the large sheath or prosthesis, and passage of the stent under fluoroscopy. In addition to the standard stent-assisted angioplasty, lithoplasty of the frequently heavily calcified vessels has proven helpful (28, 29).

We here showed that careful access planning is necessary to address the existing barriers in the iliofemoral vessels before the TAVI procedure. For this reason, from an angiological point of view, the expansion of our knowledge concerning periinterventional vascular procedures, is of immense clinical interest to improve the outcome of the patients. Ideally, a standardized perioperative treatment algorithm should be postulated, such as duplex ultrasonography of the femoral and iliac vessels and CT-scan of the aorto-iliac vessels, if necessary. An ultrasound-guided puncture is recommended to reduce the rate of access site complications (9, 10). It would also be worth considering involving the vascular Interventionalist in the decision-making process in advance, for example, as part of the interdisciplinary heart team. In any case, the large-scale prospective clinical studies with close involvement of interventional angiology would be desirable for achieving this aim.

## Conclusion

The TAVI procedure has revolutionized the treatment of aortic valve stenosis and dramatically improved the prognosis of these patients. However, it is associated with a high rate of potential peripheral complications and access the barriers that the heart team must be aware of. In addition to the advancement of devices toward smaller diameters and the introduction of new vascular closure devices, training the heart team to address these issues is a key focus. We recommend that an interventional vascular specialist becomes an integral part of the heart team.

## Data Availability Statement

The raw data supporting the conclusions of this article will be made available by the authors, without undue reservation.

## Ethics Statement

The studies involving human participants were reviewed and approved by Ethics Committee of the University Hospital Jena/the Friedrich Schiller University Jena (registration number: 4815-06/16). The patients/participants provided their written informed consent to participate in this study. Written informed consent was obtained from the individual(s) for the publication of any potentially identifiable images or data included in this article.

## Author Contributions

All authors listed have made a substantial, direct and intellectual contribution to the work, and approved it for publication.

## Conflict of Interest

The authors declare that the research was conducted in the absence of any commercial or financial relationships that could be construed as a potential conflict of interest.

## Publisher's Note

All claims expressed in this article are solely those of the authors and do not necessarily represent those of their affiliated organizations, or those of the publisher, the editors and the reviewers. Any product that may be evaluated in this article, or claim that may be made by its manufacturer, is not guaranteed or endorsed by the publisher.
